# Stability, cytotoxicity and cell uptake of water-soluble dendron–conjugated gold nanoparticles with 3, 12 and 17 nm cores†
†Electronic supplementary information (ESI) available: Additional characterization methods and procedures in addition to the data for the characterization of glutathione-capped gold nanoparticles and dendron-conjugated gold nanoparticles including FT-IR spectra (Fig. S1 and S2), UV-vis spectra (Fig. S3 and S6), TEM images (Fig. S4), MALDI-TOF/TOF spectra (Fig. S5), fluorescence spectra (Fig. S6 and S7), *In vitro* cytotoxic assay results (Fig. S9) and ICP-MS results (Tables 1 and 2). DOI: 10.1039/c5tb00608b



**DOI:** 10.1039/c5tb00608b

**Published:** 2015-07-01

**Authors:** Suprit Deol, Nisala Weerasuriya, Young-Seok Shon

**Affiliations:** a Department of Chemistry and Biochemistry , California State University , Long Beach , 1250 Bellflower Blvd. , Long Beach , California 90840 , USA . Email: ys.shon@csulb.edu

## Abstract

This article describes the synthesis of water-soluble dendron–conjugated gold nanoparticles (Den–AuNPs) with various average core sizes and the evaluation of stability, cytotoxicity, cell permeability and uptake of these materials. The characterization of Den–AuNPs using various techniques including transmission electron microscopy (TEM), matrix-assisted laser desorption/ionization-time of flight mass spectrometry (MALDI-TOF-MS), ^1^H NMR, FT-IR, and UV-vis spectroscopy confirms the dendron conjugation to the glutathione-capped gold nanoparticles (AuNPs). The stability of AuNPs and Den–AuNPs in solutions of different pH and salt concentration is determined by monitoring the changes in surface plasmon bands of gold using UV-vis spectroscopy. The stability of Den–AuNPs at different pH remained about the same compared to that of AuNPs. In comparison, the Den–AuNPs are found to be more stable than the precursor AuNPs maintaining their solubility in the aqueous solution with the salt concentration of up to 100 mM. The improved stability of Den–AuNPs suggests that the post-functionalization of thiol-capped gold nanoparticle surfaces with dendrons can further improve the physiological stability and biocompatibility of gold nanoparticle-based materials. Cytotoxicity studies of AuNPs and Den–AuNPs with and without fluorophores are also performed by examining cell viability for 3T3 fibroblasts using a MTT cell proliferation assay. The conjugation of dendrons to the AuNPs with a fluorophore is able to decrease the cytotoxicity brought about by the fluorophore. The successful uptake of Den–AuNPs in mouse fibroblast 3T3 cells shows the physiological viability of the hybrid materials.

## Abstract 2

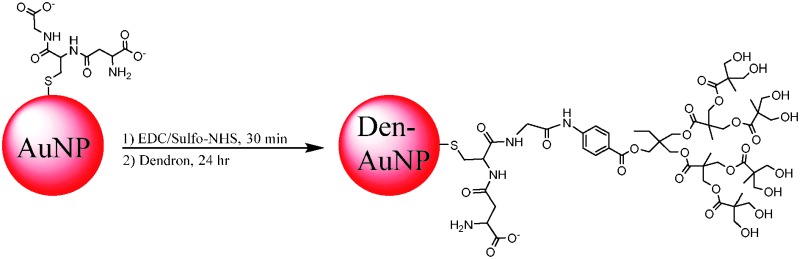
The synthesis and characterization of water-soluble dendron–conjugated gold nanoparticles (Den–AuNPs) with regard to stability, cytotoxicity and cell uptake are presented.

## 1. Introduction

The development of simple and safe ways to detect and cure diseases is considered to be one of the higher priority areas in the field of biotechnology and medical research.^1^ Recently, the advances in nanoscience and nanotechnology have greatly promoted the rapid discovery of various new systems for medical applications.^1–4^ Among various nano-systems, nanoparticles are especially ideal for medical therapies, because they are small enough to go through the capillary microcirculation but big enough for delivery *via* passive and active targeting.^5,6^ In addition, the nanoparticle platforms designed to have functional biological and synthetic molecules within a matrix would allow for entire scope including targeting, imaging and killing of cancer cells.^2–8^

Within the last decade, gold nanoparticles, which are attributed with having little toxicity to the animal and human body, have been some of the most popular choices for diverse biological and medical applications ranging from optical biomarkers to nanocarriers for cancer diagnosis and therapy.^9–15^ A feasibility to control the size and shape that govern spectral and theranostic properties made gold nanoparticles the most ideal candidates as diagnostic and therapeutic agents.^5,6,16^ Indeed, various ligand-coated gold nanoparticles have been used for receptor-mediated targeting systems (*e.g.* with antibodies against breast cancer and type 1 collagen fibrils [TGF]).^17–19^ Although currently known ligand-capped gold nanoparticles offer great potential, they are still slow to be used in clinical applications.^20^ In general, these ligand-capped gold nanoparticles do not have sufficiently high colloidal stability required for the prevention of particle agglomeration under physiological conditions. The intrinsic cytotoxicity of capping ligands surrounding nanoparticles has been another concern for clinical use. Recent studies on gold nanoparticles stabilized with specific surface ligands such as poly (ethylene glycol) (PEG-SH, 5 kDa) and glutathione (GSH) are good examples of attempts at improving the biocompatibility of ligand-coated gold nanoparticles.^3^

Dendrimers are another organic species that are popularly used for the stabilization of metal nanoparticles.^21–23^ The positive influence of dendrimers on nanoparticle stability and biocompatibility was the main reason for the increased interests in dendrimer–gold nanoparticle composite systems for nano-bio applications.^21,22^ Even dendrimers by themselves have been frequently used for biological applications in drug delivery and as biomarkers.^24,25^ With their well-defined and highly tunable globular structures, dendrimers can be regulated as a function of their size, shape, surface chemistry and interior void space, which are conjugated with drug molecules, signaling groups, or biocompatibility groups.^26,27^ Dendrimer surfaces may be functionally designed to enhance or resist trans-cellular, epithelial or vascular biopermeability. The most important features of dendrimers are their low toxicity, acceptable excretionary pathways and non-immunogenic characteristics. Due to these reasons, the U.S. FDA has approved dendrimers for human clinical testing.^25^

Dendrimer–encapsulated nanoparticles (DENs) are the composite system which have recently gained much interest.^28–31^ Some of the advantages include encapsulation of gold nanoparticles in the central core for enhanced stability, size control based on the number of generations, and multiple possible functaionalizations.^32^ Because nanoparticles are generated inside the cavities of dendrimers by the reduction of metal precursors (salts), DENs face some limitations over the structural integrity and composition. First, dendrimers need to hold high concentration of metal salts in the interior, limiting the eligible host dendrimers to only a few different types (*e.g.* PAMAM, PPI). Second, the size of encapsulated nanoparticles is clearly limited to the size of dendrimers. It requires extremely large dendrimers to synthesize DENs with nanoparticles larger than 5–10 nm. Third, most importantly, the encapsulated nanoparticles are easily extracted from dendrimers by organic ligands. For example, simple alkanethiols were able to extract metal NPs from PAMAM dendrimers after simple mixing and shaking.^33^

The use of dendrons as stabilizing ligands around gold nanoparticle cores would present several advantages over DENs including: (i) enhanced stability *via* covalent bonds between dendron ligands and nanoparticle cores, (ii) the formation of dendronized nanoparticles with controlled shell thickness, and (iii) the encapsulation of nanoparticles with various sizes and shapes.^34,35^ Three different synthetic methodologies have been applied for the preparation of dendron–conjugated gold nanoparticles (Den–AuNPs): the direct method, ligand-exchange method, and convergent reaction method.^34^ The Shon group has developed the convergent method, which is a versatile synthetic process to efficiently build dendritic frameworks of specific size and composition around a gold nanoparticle core by post-conjugation of dendrons to ligand-capped gold nanoparticles.^36–39^ With covalently attached biocompatible dendron shells, the Den–AuNPs may resist aggregation in biological fluids better than the more conventional ligand-capped gold nanoparticles. Therefore, the conjugation with dendrons to the pre-formed functionalized gold nanoparticles would provide another means of improving biocompatability of gold nanoparticles.

The ability to link tracking fluorophores within the open framework of these dendritic coverings rather than on the surface of the coating can also lower their cellular toxicity. It has been known that the cytotoxicity of gold nanoparticles increases to an unsafe level for use under biological conditions when fluorophores are bound and exposed. Direct application of fluorophores in biological systems has also been handicapped by their poor stability (aggregation) in solution and by the complex dependence of their fluorescence intensity on concentration.^40^ Attaching the fluorophores in the interior of DEN–AuNPs covalently will further increase their integrity and biostability. The toxicity of fluorophores to the normal cells, in general, is also expected to decrease due to the presence of dendron shells around the fluorophore resulting in the segregation from the cells.

This article describes a strategy to synthesize water-soluble, stable and biocompatible dendron–conjugated gold nanoparticles as multifunctional diagnostics agents. The bis-MPA dendrons (2,2-bis(hydroxyl-methyl)propionic acid dendrons) are chosen, because the study involving the stability and biocompatibility of a library of polyester dendrimers demonstrated that the bis-MPA dendrimer is degradable and non-cytotoxic to human cell lines and primary cells.^22^ The objective of this work is to first synthesize and characterize Den–AuNPs with core sizes of 3, 12 and 17 nm, and to attach fluorophores between the gold surface and the dendrons. The second objective is to determine the stability of the AuNPs and Den–AuNPs over a range of pH and salt concentrations. The third objective is to determine the cytotoxicity of the AuNPs and Den–AuNPs towards NIH-3T3 cells by performing cell viability assays. The second and third objectives aim at comparing the effects of surface chemistry (AuNPs *vs.* Den–AuNPs) as well as the core size.

## 2. Experimental

### 2.1. Materials and methods

The necessary chemicals and reagents were purchased from the following suppliers, and used as provided unless stated. Hydrogen tetrachloroaurate(iii) trihydrate and trisodium citrate dibasic were from Acros Organics. Tissue culture treated multiwell plates were from BD Falcon. Deuterium oxide and methanol d-4 were from Cambridge Isotope Laboratories, Inc. Fetal bovine serum (FBS), Dulbecco's modification of Eagle's medium (DMEM), Dulbecco's phosphate-buffered saline (DPBS) without calcium and magnesium and trypsin:EDTA were from Cellgro. Dodecyl sodium sulfate (SDS) and *N*,*N*-dimethylformamide were from Eastman. Osmium tetraoxide, glutaraldehyde, propylene oxide, araldite 502 kit, lead citrate and uranyl acetate were from Electron Microscopy Sciences. Reduced glutathione, sodium borohydride, sodium phosphate monobasic anhydrous, sodium phosphate dibasic dihydrate, glass coverslips and ethanol (200 proof) were from Fisher Scientific. 7-Amino-4-methyl-3-coumarinylacetic acid (AMCA) was from Fluka Analytical. Penicillin, streptomycin and l-glutamine were from Lonza. The 0.22 Micron PES membrane sterile filters were form Millex. The bis-MPA dendron (PFd-G3-ArNH_2_-OH) was from Polymer Factory. α-Cyano-4-hydroxycinnamic acid (CHCA) was from ProteoChem. Sodium cyanide, trypan blue and thiazolyl blue tetrazolium dye (MTT) were from Sigma Aldrich. Spectra/Por 7 dialysis tubing was from Spectrum Laboratories, Inc. 1-(3-Dimethylaminopropyl)-3-ethylcarbodiimide hydrochloride (EDC) was from TCI America. The 200 Mesh copper formvar/carbon coated TEM grids were from Ted Pella, Inc. *N*-Hydroxy-sulfosuccinimide (Sulfo-NHS) and DyLight 755 NHS ester were from Thermo Scientific. NIH/3T3 (ATCC CRL-1658) mouse embryonic fibroblasts were donated. All water was obtained from a Milli-Q water purification system.

#### 2.1.1. Synthesis of 3 nm gold nanoparticles

Glutathione-capped gold nanoparticles (AuNPs) with an average core size of 3 nm were synthesized using the published method.^41^ All glassware was cleaned with aqua regia (3 : 1 concentrated hydrochloric acid: concentrated nitric acid) and rinsed with copious amounts of water. First, 1 mmol of glutathione was added to a 250 mL round bottom flask containing 50 mL of HAuCl_4_ in 60% methanol (20 mM) while stirring vigorously. After 40 min, 40 mL of sodium borohydride (0.125 M) was added slowly, and then the flask was quickly placed in a 50 °C water bath for one hour while maintaining the vigorous stirring. The resulting gold nanoparticles were concentrated by rotary evaporation, purified by dialysis (8–10 kDa MWCO membrane) against water for 3 days, and then freeze dried.

#### 2.1.2. Synthesis of 12 and 17 nm gold nanoparticles

The synthesis of citrate-stabilized 12 nm gold nanoparticles was done according to the previously published methods.^42,43^ A 200 mL aq. solution of HAuCl_4_ (1 mM) was refluxed for 15 min in a 500 mL round bottom flask with rapid stirring. A 20 mL trisodium citrate (38.8 mM) was quickly added to the boiling HAuCl_4_ solution, and allowed to reflux for an additional 10 min. The 17 nm citrate-stabilized gold nanoparticles were also synthesized using a previously published procedure.^44^ Briefly, 95 mL of HAuCl_4_ (0.29 mM) was refluxed in a 250 mL round bottom flask for 10 min. While stirring vigorously, 5 mL of trisodium citrate (34 mM) was quickly added, resulting in a color change for yellow to red. This was refluxed for an additional 15 min. Glutathione-capped gold nanoparticles were obtained by ligand exchange of the 12 and 17 nm citrate gold nanoparticles according to a previously published method with slight modification (Fig. S1, ESI†).^45,46^ A 100 mg mL^–1^ aq. glutathione solution was freshly prepared and 150 μL of this solution was added to 10 mL aq. solution of 12 nm citrate-stabilized gold nanoparticles and 12 mL aq. solution of 17 nm citrate-stabilized gold nanoparticles. The gold nanoparticles were mixed and allowed to precipitate for one hour at room temperature. The black precipitate was pelleted by brief centrifugation (1000 × g) and was washed three times with water. The precipitated AuNPs were reconstituted in 10 mM dibasic sodium phosphate, pH 7.2.

#### 2.1.3. Dendron conjugation to gold nanoparticles

**Scheme 1:**
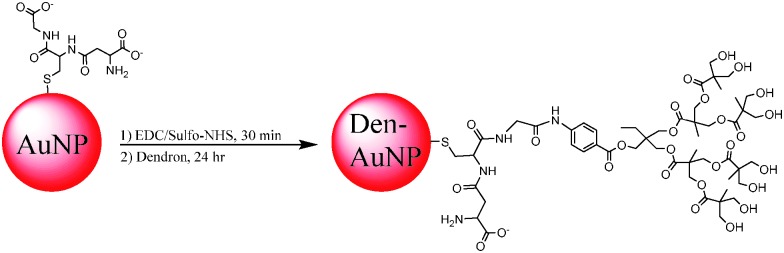
Synthesis of dendron–conjugated gold nanoparticles (Den–AuNPs).

The coupling of bis-MPA dendrons (PFd-G3-ArNH_2_-OH) to glutathione-capped gold nanoparticles was done with 1-ethyl-3-(3-dimethyl-aminopropyl) carbodiimide (EDC) and *N*-hydroxysulfo-succinimide (sulfo-NHS) as shown in Scheme 1. Dendron conjugation for 3 nm AuNPs was accomplished by dissolving 30 mg of gold nanoparticles in 3 mL of 10 mM sodium phosphate buffer (pH 7.2), and adding 496 μL of aq. EDC (11.83 mg mL^–1^) solution and 2303 μL of aq. sulfo-NHS (2.83 mg mL^–1^) solution and stirring for 30 min at room temperature. This was followed by the addition of 5 mL of aq. dendron (3.14 mg mL^–1^) solution and the mixture was allowed to react for 12 h with stirring. The 3 nm Den–AuNPs were purified by dialysis against 10 mM sodium phosphate buffer (pH 7.2) and then freeze dried for 3 days. The products were analyzed by UV-vis, FT-IR, and ^1^H-NMR spectroscopy.

Dendron conjugation for 12 and 17 nm AuNPs was done in a similar fashion. To 1 mL of aq. gold nanoparticle solution, 15 μL of aq. EDC (1 mg mL^–1^) solution and 15 μL of aq. sulfo-NHS (1 mg mL^–1^) solution were added and mixed for 1 h. After that, 200 μL of aq. dendron (0.5 mg mL^–1^) solution was added and mixed for 24 h.

#### 2.1.4. AMCA conjugation

**Scheme 2:**
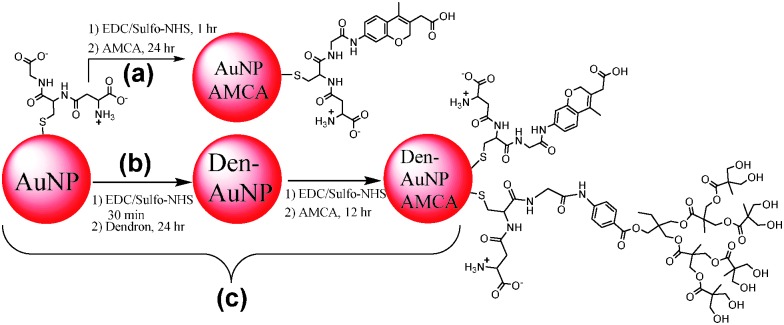
AMCA conjugation to glutathione-capped gold nanoparticles (AuNPs) and dendron–conjugated gold nanoparticles (Den–AuNPs).

AMCA was conjugated to both 3 nm AuNPs and Den–AuNPs. First, 2.75 mL of AuNP solution (10.9 mg mL^–1^) was mixed with 1.07 mL of aq. EDC (5.03 mg mL^–1^) solution and 1.176 mL of aq. sulfo-NHS (5.1 mg mL^–1^) solution for 1 hour to activate carboxyl groups on glutathione for subsequent reaction. For conjugation to AuNPs (Scheme 2a), 578 μL of aq. AMCA (1.95 mg mL^–1^) solution was added and stirred for 24 hours. For conjugation to Den–AuNPs (scheme 2c), 10 mL of dendron solution (0.946 mg mL^–1^) was reacted for 12 hours, and then 750 μL of aq. EDC (5.03 mg mL^–1^) solution, 500 μL of aq. sulfo-NHS (5.1 mg mL^–1^) solution, and 289 μL of aq. AMCA (1.95 mg mL^–1^) solution were added and mixed for another 12 hours. Both reactions were purified by dialysis against nanopure water, and then lyophilized. The products were analyzed by fluorescence and ^1^H-NMR spectroscopy.

#### 2.1.5. Conjugation of DyLight 755 NHS ester

DyLight 755 amine-reactive dye was conjugated to 3, 12 and 17 nm AuNPs and Den–AuNPs. The dye (1 mg) was first dissolved in 1 mL of water and mixed by vortexing, resulting in a teal green color. For conjugation, 1 mL of 3, 12 and 17 nm AuNP and Den–AuNP solutions was mixed with 200, 150, and 100 μL of dye, respectively. The dye was allowed to react overnight with shaking. Unbound dye was removed by dialysis against water with an 8–10 kDa MWCO dialysis membrane.

#### 2.1.6. Stability testings

The stability of AuNPs and Den–AuNPs was tested at varying pH and salt concentrations. For salt stability, AuNPs and Den–AuNPs were diluted with different sodium chloride solutions ranging from 0–200 mM and the absorbance was measured from 400–800 nm using UV-vis spectroscopy. The stability of AuNPs and Den–AuNPs at different pH was determined by diluting samples in sodium phosphate buffer with desired pH and the subsequent analyses by UV-vis spectroscopy.

### 2.2. Cell culture

NIH/3T3 (ATCC CRL-1658) mouse embryonic fibroblasts were cultured according to the ATCC protocol.^47^ They were maintained in Dulbecco's modified eagle medium (DMEM) supplemented with 10% fetal bovine serum (heat inactivated),^48^ 1% penicillin, 1% streptomycin, and 1% l-glutamine in a humidified incubator (95% relative humidity, 5% CO_2_) at 37 °C. They were subcultured twice a week at 70–80% confluency. Briefly, the spent culture medium was discarded, the cells were washed twice with Dulbecco's phosphate buffered saline (dPBS). dPBS was discarded and 2 mL of trypsin:EDTA (0.25%:0.53 mM) was added and the flask was placed in the incubator for 5 min to detach the cells. The trypsin was neutralized by adding 4 mL of the culture medium, and a new T75 flask was inoculated with 3 × 10^5^ cells per mL.

#### 2.2.1. Cell viability assays

Cell viability was determined by MTT assay (Promega). Cells were counted with trypan blue (1 : 1 dilution) on a hemocytometer, and only live cells were counted for calculating concentration. Cells were diluted with culture medium, and 24 well plates were seeded with 5000 cells per well (500 μL) and incubated for 24 hours to allow cells to adhere. The exhausted media were aspirated, and replaced with 500 μL of fresh media containing 10% (v/v) test samples, and incubated for 24 hours. The media containing test samples were gently aspirated, and 500 μL of media containing MTT (1 mg mL^–1^ final concentration) was added and incubated for 4 hours. The media were then carefully aspirated and 500 μL of a 20% SDS/50% DMF, pH 4.7 solution was added and incubated overnight to solubilize the formazan crystals.^49^ The next day, the plates were shaken for a few minutes to homogenize the well contents, and the absorbance was measured at 570 nm on Thermo Varioskan.

#### 2.2.2. Cell uptake

Sterile glass coverslips (washed with aqua regia) were placed in a 6 well plate and 3 mL of 1 × 10^5^ cells per mL were added. The cells were incubated for 10 hours, and then 300 μL of Den–AuNPs were added and incubated for 6 hours. The culture medium was aspirated and replaced with 3–5 mL of fixing solution (1% glutaraldehyde in 1× Sorensen's phosphate buffer, pH 7.3) for 30 min. The fixing process was initiated at 37 °C and then cells were placed on ice after approximately 5 min. The fixing solution was removed and the cells were rinsed with ice-cold Sorensen's phosphate buffer three times. Next, the cells were fixed with 3 mL of 1% osmium tetroxide in Sorensen's Phosphate Buffer for 30 min. The 1% osmium tetroxide solution was removed and cells were rinsed with ice-cold Sorensen's phosphate buffer three times (approximately 15 min per rinse). The cells were dehydrated quickly with 30, 50, 75, 95 and 100% cold ethanol gradients. The cells were subjected to 100% ethanol three times, propylene oxide three times, and then a 50/50 mixture of propylene oxide/araldite 502 resin (with activator), and then coverslips were placed cell-side down on araldite 502 resin. The resin was cured at 60 °C for 3 days under slight vacuum.

The glass coverslips were removed by freezing in liquid nitrogen. The blocks were sanded to reduce the thickness, and small pieces were chipped out with a razor blade and hammer. These were then adhered to flat stud blocks with superglue, and trimmed for sectioning. “Gold” sections (∼75–80 nm thickness) were obtained using a thermal advance ultramicrotome (LKB Ultrotome Nova). The sections were wafted with toluene and collected on clean 200-mesh copper grids. The sections were post-stained with uranyl acetate for 3.5 min, and then with lead citrate for 2.5 min. The sections were imaged by TEM.

## 3. Results and discussion

### 3.1. Synthesis of dendron–conjugated gold nanoparticles with various core sizes

**Fig. 1:**
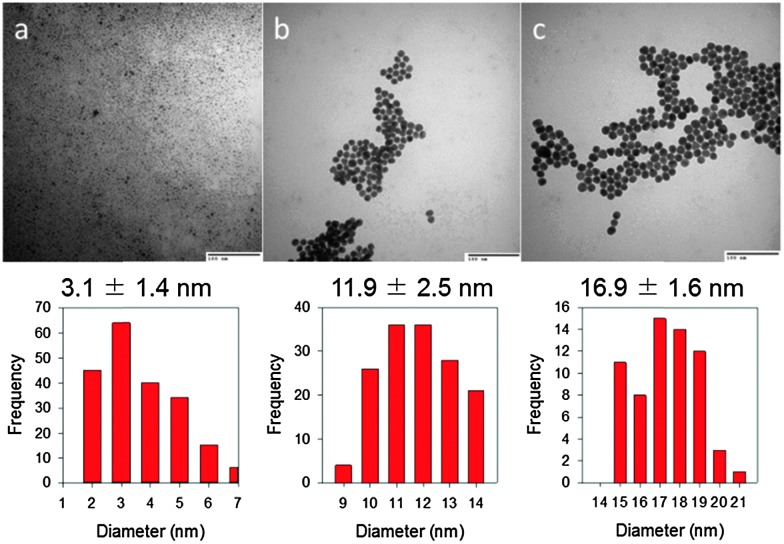
TEM images and histograms of glutathione-capped gold nanoparticles (AuNPs): (a) 3.1 ± 1.4 nm (see also Fig. S4, ESI†), (b) 11.9 ± 2.5 nm and (c) 16.9 ± 1.6 nm. The scale bars in all three TEM images are 100 nm.

**Fig. 2:**
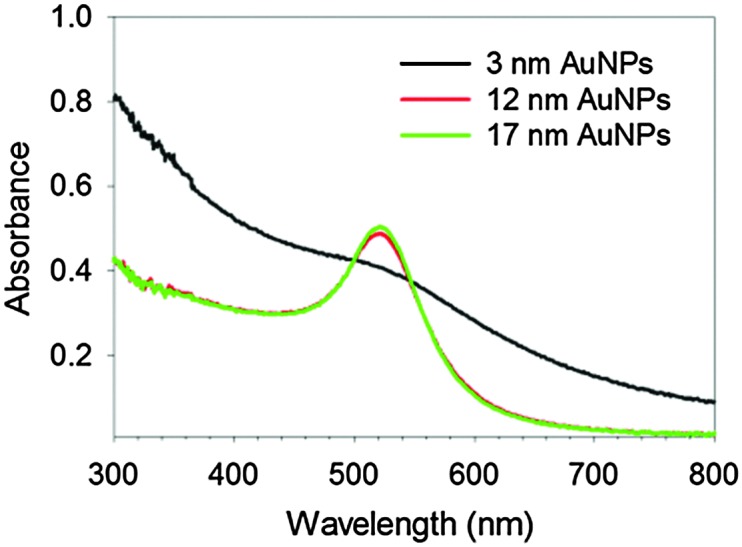
UV-vis spectra of 3, 12 and 17 nm AuNPs dissolved in PBS buffer solution. The concentration of AuNPs is ∼0.1 mg mL^–1^.

Glutathione-capped gold nanoparticles were synthesized either by using the modified Brust methods (3 nm) directly or the Turkevich methods followed by ligand exchange (12 and 17 nm).^41–44^ The synthesis of nanoparticles with different sizes was verified by TEM and UV-vis spectroscopy. The TEM images showed spherical gold nanoparticles for each case and the calculated average core diameters were 3.1 ± 1.4, 11.9 ± 2.5 and 16.9 ± 1.6 nm (Fig. 1a–c). UV-vis spectra of 3 nm AuNPs had a very weak surface plasmon (SP) band at 518 nm, which is a characteristic of small gold nanoparticles, while those of 12 and 17 nm AuNPs had strong SP bands at 521 and 522 nm, respectively (Fig. 2). The wavelength of SP bands without any red-shift indicates that these AuNPs are free of aggregation and coarsening.

The Den–AuNPs were synthesized by conjugating dendrons to AuNPs by the amide coupling reaction of COOH groups in glutathione ligands with NH_2_ groups in dendrons (PFd-G3-ArNH_2_-OH) in the presence of coupling reagents (EDC and sulfonate-NHS).^50^ The 3 nm Den–AuNPs could maintain a high solubility in PBS buffer solution (pH = 7.2) for an extended period and could be isolated and redissolved in the buffer solution.

**Fig. 3:**
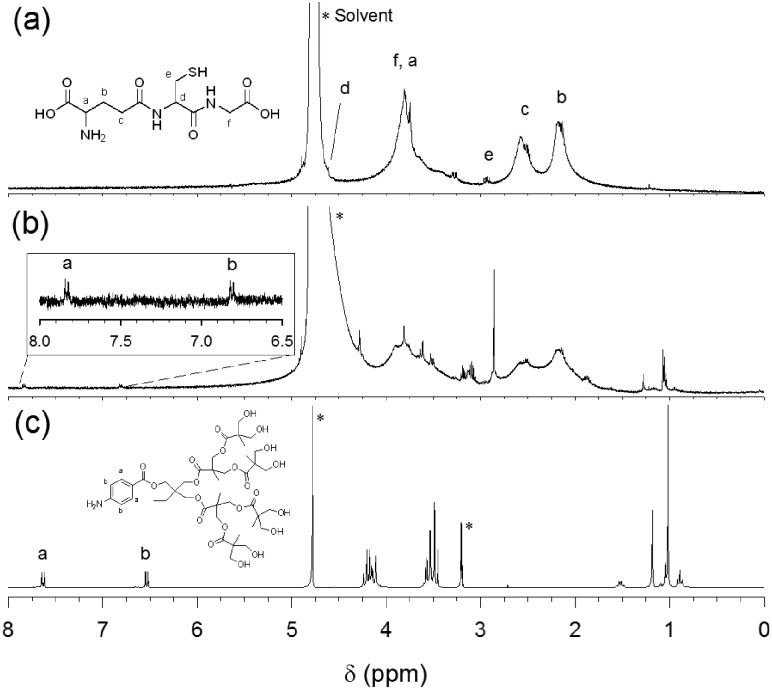
^1^H NMR spectra of (a) 3 nm AuNPs in D_2_O, (b) 3 nm Den–AuNPs in D_2_O and (c) dendrons in MeOD. Peak labels are the same for spectra b and c. The signals with * are resulted from the presence of solvents (H_2_O and CH_3_OH).

The analysis of 3 nm Den–AuNPs by ^1^H-NMR and FT-IR spectroscopy showed the chemical changes of surface ligands after the conjugation of dendrons. The ^1^H-NMR spectra of AuNPs, Den–AuNPs and dendrons are displayed in Fig. 3. The spectrum of AuNPs (Fig. 3a) has broad peaks with extensive overlaps. This broadening is accounted for the spin–spin relaxation (T2-broadening) or the existence of a distribution of chemical shifts caused by the differences in the Au–S binding sites (terraces, edges, vertices).^51^ The same peak-broadening effect was observed for the monolayers of alkanethiolate-protected nanoparticles generated from alkanethiols.^51^ The spectrum of Den–AuNPs (Fig. 3b) displays new signals that were not present in the spectrum of AuNPs (Fig. 3a), with the main focus on two proton signals in the aromatic region (*δ*_peak a_: 7.8, *δ*_peak b_: 6.8) and the multiple signals in the upfield methyl/methylene region (*δ*_peak CH_3__: 0.9–1.3). This strongly suggested the conjugation of dendrons to AuNPs. The ratio of integration values for GSH to dendron was used to estimate ∼10 dendron molecules per each particle.

The 3 nm Den–AuNPs were also analyzed by FT-IR spectroscopy, which showed shifts in carbonyl bands at ∼1600–1750 cm^–1^ after the conjugation of dendrons (Fig. S2, ESI†). The formation of amide bonds after a successful coupling reaction between glutathione and dendron is indicated by the presence of a strong absorption at ∼1650 cm^–1^ in place of carbonyl bands from either COOH (1710 cm^–1^) or COO^–^ (1580 cm^–1^) groups of glutathione. IR spectra of Den–AuNPs also showed a decrease in the intensity of O–H absorption at 2500–3500 cm^–1^ indicating the conversion of COOH into amide groups. This result is another supporting evidence for the conjugation of dendrons to AuNPs by the coupling reaction.

Although the absorbance at 290 nm from the dendrons was too small to be clearly seen in the UV-vis spectra of the 3 nm Den–AuNPs (Fig. S3, ESI†), the presence of attached dendrons was further confirmed by the MALDI-TOF/TOF mass spectra of dendrons and 3 nm Den–AuNPs that are plotted in Fig. S5 (ESI†). The 3 nm Den–AuNPs were treated with sodium cyanide to form an Au–CN complex, liberating surface ligands. The mass spectra of 3 nm Den–AuNPs have a major *m*/*z* peak at 466.40 (Fig. S5, ESI†). This peak was assigned as a fragment of dendrons formed during the sodium cyanide treatment and confirmed the presence of dendrons on the surface of AuNPs. The mass spectrum of dendrons itself is shown as a comparison in Fig. S5a (ESI†), in which the *m*/*z* of 972.18 corresponds to the mass of dendrons plus sodium ions. The UV-vis spectra of 3 nm Den–AuNPs also showed a slightly broadened SP band after the conjugation of dendrons (Fig. S3, ESI†). The intensity of a SP band typically reflects relative average core sizes of nanoparticles and/or interactions between surrounding ligands and the surface.

The UV-vis spectra of 12 and 17 nm Den–AuNPs during dialysis are shown in Fig. S6 (ESI†). The spectra show that the two absorbance peaks corresponding to dendrons (233 and 290 nm) decrease with subsequent buffer changes, and are still visible at the end of dialysis without any further change. The SP bands remained at around 520 nm for both Den–AuNPs without any noticeable shift, which indicates the absence of evident aggregation. The dialysis time could be shortened to 3 hours by using a number of dialysis membranes and 5000× dialysate volume every hour. This was critical because the longer dialysis caused some flocculation of both 10 and 17 nm Den–AuNPs.

**Fig. 4:**
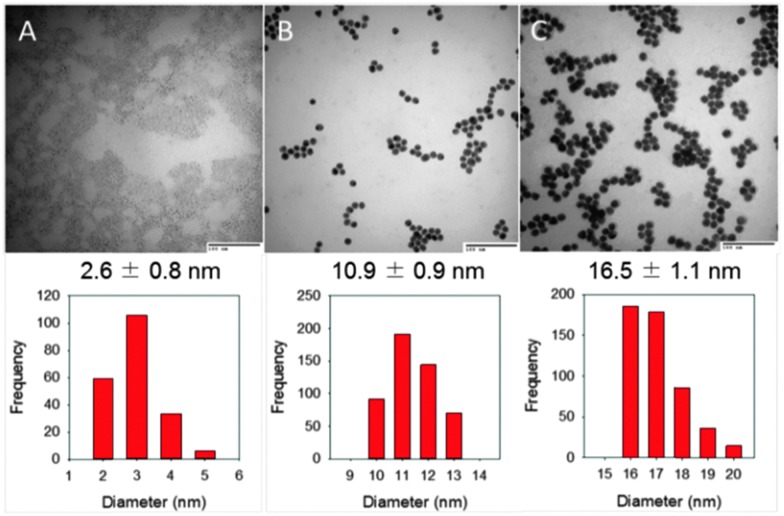
TEM images and histograms of Den–AuNPs: (A) 2.6 ± 0.8 nm (see also Fig. S4, ESI†), (B) 10.9 ± 0.9 nm and (C) 16.5 ± 1.1 nm. The scale bars in all three TEM images are 100 nm.

TEM images of Den–AuNPs are shown in Fig. 4 and suggested that the particles were well dispersed in buffer solutions without forming much fused particle domains. The Den–AuNPs were still in a spherical shape as observed from AuNPs. TEM histogram analyses confirmed that the size of Au nanoparticle core was mostly preserved through the dendron attachment processes without the occurrence of core coalescence. The calculated diameters for the Den–AuNPs are 2.6 ± 0.8, 10.9 ± 0.9 and 16.5 ± 1.1 nm. This result proves that our approach is indeed useful for the synthesis of Den–AuNPs with controlled core sizes up to 20 nm. In conjunction with UV-vis results, therefore, the slight changes in the features and intensities of SP bands of Den–AuNPs support the dendron attachments, considering their comparable average core sizes after the coupling reaction as shown in TEM studies.

### 3.2. Incorporation of fluorophores into dendron–conjugated gold nanoparticles

**Fig. 5:**
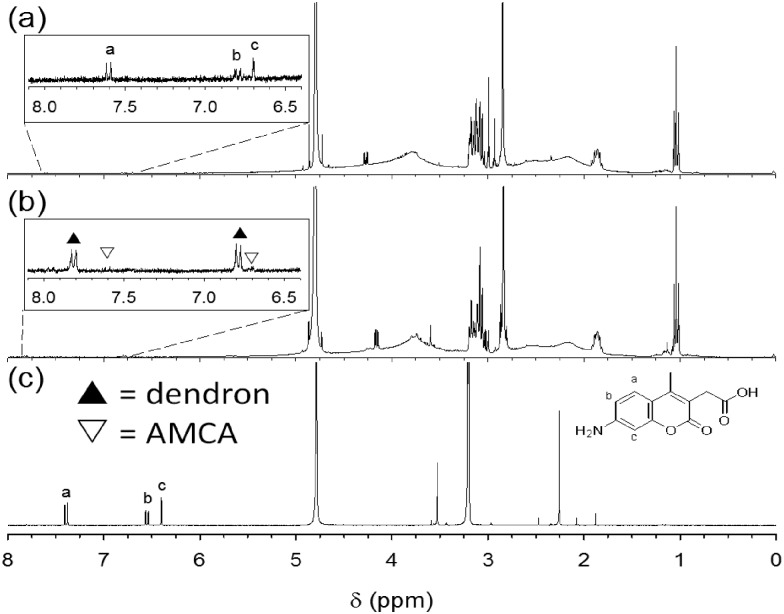
^1^H NMR spectra of (a) 3 nm AuNPs–AMCA in D_2_O, (b) 3 nm Den–AuNPs–AMCA in D_2_O and (c) AMCA in MeOD. Peak labels are the same for spectra a and c; in spectrum b, aromatic protons from dendrons and AMCA are labeled with black and white asterisks, respectively.

Dendritic encapsulation of a functional molecule provides a site isolation, which mimics principles from biomaterials because dendritic scaffolds can provide the segregation of external and internal functionality.^24^ The conjugation of the fluorophore, 7-amino-4-methyl-3-coumarinylacetic acid (AMCA), to AuNPs and Den–AuNPs was verified by ^1^H NMR (Fig. 5). The presence of signals a–c in the aromatic region of Fig. 5a corresponds to the aromatic protons of AMCA. The spectrum in Fig. 5b is of the 3 nm Den–AuNPs conjugated with AMCA (Den–AuNPs–AMCA), and the signals arising from aromatic protons from dendrons and AMCA are labeled. It is estimated that there are two AMCA molecules per Den–AuNPs–AMCA, based on a ratio of integration values.

However, the AMCA attached to AuNPs with different core sizes (3, 12 and 17 nm) suffered from a complete quenching of fluorescence by a gold core (Fig. S7, ESI†).^52,53^ This is because the emission wavelength of AMCA at ∼450 nm is close to the SP band of gold at ∼520 nm. Increasing the distance between the gold surface and AMCA is one way to overcome the quenching of fluorescence, but it was impractical for our approach requiring the placement of fluorophores under the dendron branches. Several groups have reported fluorescence or even enhanced fluorescence by using near-infrared (NIR) fluorophores with different spacer lengths.^52^ The results suggested that for the gold nanoparticles of these core sizes the coupling of NIR fluorophores with emission wavelengths between 700 and 800 nm might be necessary to avoid such quenching problems.

The incorporation of DyLight 755 on the surface of AuNPs and in the pore of Den–AuNPs was attempted using the similar coupling strategy utilized for dendron coupling and other fluorophore attachments. The fluorescence spectra of AuNPs-DyLight 755 and Den–AuNPs-DyLight 755 are plotted in Fig. S8 (ESI†). The DyLight 755 NIR fluorophore exhibited strong emission properties even after attachment to gold nanoparticles with larger core sizes and was free of quenching problems previously encountered for AMCA (Fig. S8a, ESI†). There is a small difference in peak emission wavelengths, which is thought to be the result of a different microenvironment created by the dendrons (Fig. S8b, ESI†).

### 3.3. Stability of gold nanoparticles

**Fig. 6:**
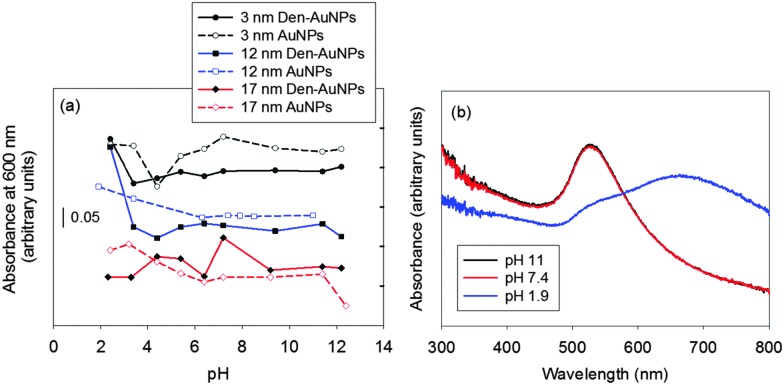
The pH stability results of AuNPs and Den–AuNPs with different core sizes: (a) absorbance at 600 nm at different pH and (b) UV-vis spectra of 12 nm AuNPs at selected pHs are displayed to show the increase in absorbance at 600 nm and the red-shifts of SP bands resulting from aggregations.

The stability of Den–AuNPs and AuNPs in different pHs and salt concentrations was studied using UV-vis spectroscopy to confirm the suitability of Den–AuNPs in future application such as a direct *in vivo* animal testing. Aggregated particles tend to have broader SP bands for gold, with *λ*_max_ shifted to a higher wavelength (Fig. 6b). Therefore, the aggregation of gold nanoparticles typically results in color changes from red to purple, to blue, and even to blue-black, which allows aggregation to be detected visually. The analysis of UV-vis spectra of AuNPs at different pH values showed that the larger 12 and 17 nm AuNPs are only stable at the pHs greater than ∼6. Lower pHs caused irreversible particle aggregation and an increase in the absorbance at 600 nm (chosen for observation of small changes) for these larger AuNPs (Fig. 6a). In comparison, the smaller 3 nm AuNPs maintained good stabilities even at the lower pHs showing no increase in the absorbance. The stability of Den–AuNPs with different sizes remained about the same (stable at the pHs as low as ∼4) compared to that of AuNPs.

**Fig. 7:**
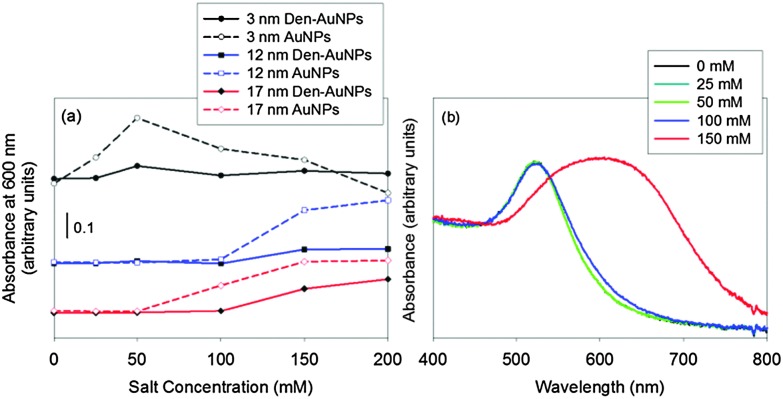
The salt stability results of AuNPs and Den–AuNPs with different core sizes: (a) absorbance at 600 nm of samples with different salt (NaCl) concentrations (*X*-axis as final concentrations) and (b) UV-vis spectra of 17 nm Den–AuNPs at different salt concentrations.

The salt stability of Den–AuNPs was also determined to be slightly higher than that of AuNPs for all sizes (Fig. 7). The UV-vis spectra in Fig. 7b show an example, the broadening and red-shift of the SP band of 17 nm Den–AuNPs at the higher salt concentration, which was due to the agglomeration of nanoparticles. The 3 nm AuNPs have even started to precipitate out of solution at the concentration above 25–50 mM NaCl. The spectral evidence of precipitation could be observed from the steep decrease in absorbance at 600 nm at higher salt concentrations. In comparison, the 3 nm Den–AuNPs did not exhibit any change in the SP band of gold implicating the positive influence of dendrons on the salt stability of AuNPs. Both the UV-vis spectra of 12 and 17 nm AuNPs after the salt treatments indicated that the AuNPs have started to aggregate at the NaCl concentration of 50 mM and above. Den–AuNPs, however, have only begun aggregating in the presence of more than 100 mM NaCl and the increase in absorbance for Den–AuNPs was less steep than that of AuNPs.

The improved stability of Den–AuNPs in solutions of higher salt concentrations indicated that the post-functionalization of nanoparticle surfaces with a few dendrons could help with controlling the physiological stability of gold nanoparticle-based materials. It is likely due to the fact that the presence of large dendron ligands further extended from the nanoparticle surface results in better protection of nanoparticle cores from the ionic disruptions.

Studies to reverse self-induced aggregation of AuNPs in higher salt concentrations were also performed. It was found that the addition of EDTA solution (pH 8.0) rapidly reversed salt-induced aggregation after the excess salts were removed by dialysis. The same reverse aggregation was also observed for the treatment of sodium citrate. The results suggested the presence of exposed gold sites on the glutathione monolayer, to which negatively charged molecules like EDTA or sodium citrate can adsorb. The adsorption of EDTA or citrate would lead to electrostatic repulsion of AuNPs, and reverse aggregation.

### 3.4. Evaluation of cytotoxicity and distribution of nanoparticles in cultured cells

**Fig. 8:**
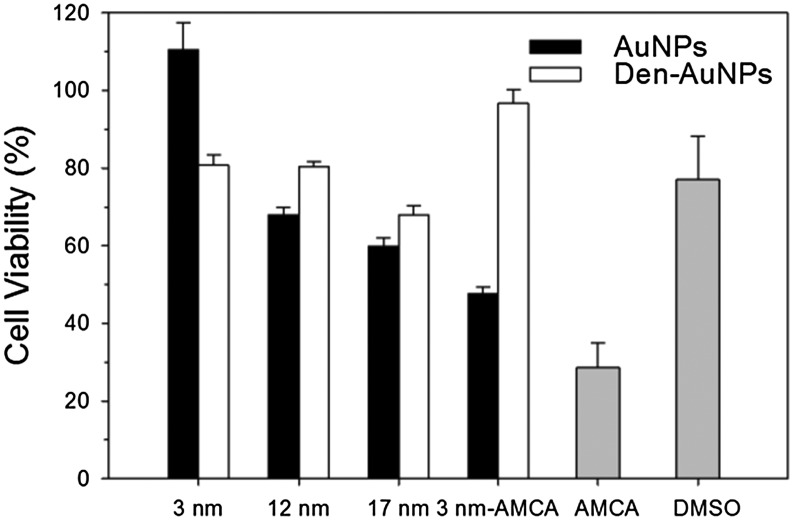
*In vitro* cytotoxic assay of AuNPs and Den–AuNPs with different core sizes, 3 nm AMCA functionalized AuNPs and Den–AuNPs, AMCA in DMSO, and DMSO (solvent control). Percentage of cell viability is with respect to solvent control (10 mM sodium phosphate buffer, pH 7.2).

Cytotoxicity studies of fluorophores (AMCA and DyLight-755), AuNPs, Den–AuNPs and fluorophore-functionalized AuNPs and Den–AuNPs were performed by examining cell viability for NIH-3T3 mouse fibroblasts using a MTT cell proliferation assay.^54^ Cell viability tests demonstrated that the cytotoxicity of AMCA by itself in DMSO was relatively high as shown in Fig. 8.^55^ The toxicity of AMCA (0.1 mg mL^–1^) in DMSO (0.1% v/v final) was about twice that of DMSO alone. The results also suggested that the cell viability of 3 nm Den–AuNPs–AMCA was nearly twice that of 3 nm AuNPs–AMCA, thus confirming our hypothesis that dendron attachment would decrease toxicity of fluorophores and enhance cell viability. This is because the encapsulation of AMCA underneath conjugated dendrons on AuNPs would segregate fluorophores from directly contacting the cells. The cell viability results indicated that as the size of AuNPs increases the cell viability of particles decreases. The cytotoxicity of smaller gold nanoparticles ranging between 1 and 20 nm is reported to be governed by the type of surrounding organic ligands rather than the sizes of nanoparticles and it is also cell specific.^56^ Therefore, the slightly decreased cell viability of larger 12 and 17 nm AuNPs is most likely caused by the negative influence of nanoparticle aggregates in the mouse fibroblasts after uptake. It was also found that the dendron attachments to larger colloidal AuNPs tend to slightly increase the cell viability due to the enhanced stability. The reason for a slight decrease in cell viability for 3 nm Den–AuNPs compared to those of 3 nm AuNPs and Den–AuNPs–AMCA is currently not understood. However, the cell viability results for all three nanoparticles are high (>80% cell viability), of which the values are accepted as nontoxic to the cells.^56^ It should be noted that the sample volume was constant, but the nanoparticle concentrations of AuNPs and Den–AuNPs with different core sizes were varied. The concentration of gold was quantified by ICP-MS (Table S1, ESI†), which showed higher concentrations for 3 nm AuNPs and Den–AuNPs compared to other particles with larger core sizes. This condition minimizes the influence of overall nanoparticle concentration on cytotoxicity, because a higher concentrated solution of smaller nanoparticles showed better cell viability and a lower concentrated solution of larger nanoparticles showed lower cell viability. For the nanoparticles with the same sizes (3 nm AuNPs, Den–AuNPs and Den–AuNPs–AMCA), the actual concentration of solution was estimated to be relatively similar. When the NIR fluorophore, Dylight-755, was used instead of AMCA, it was found that Dylight-755 did not have any significant influence over the cell viability whether they are attached to AuNPs and Den–AuNPs or they are free of any support (Fig. S9, ESI†).

**Fig. 9:**
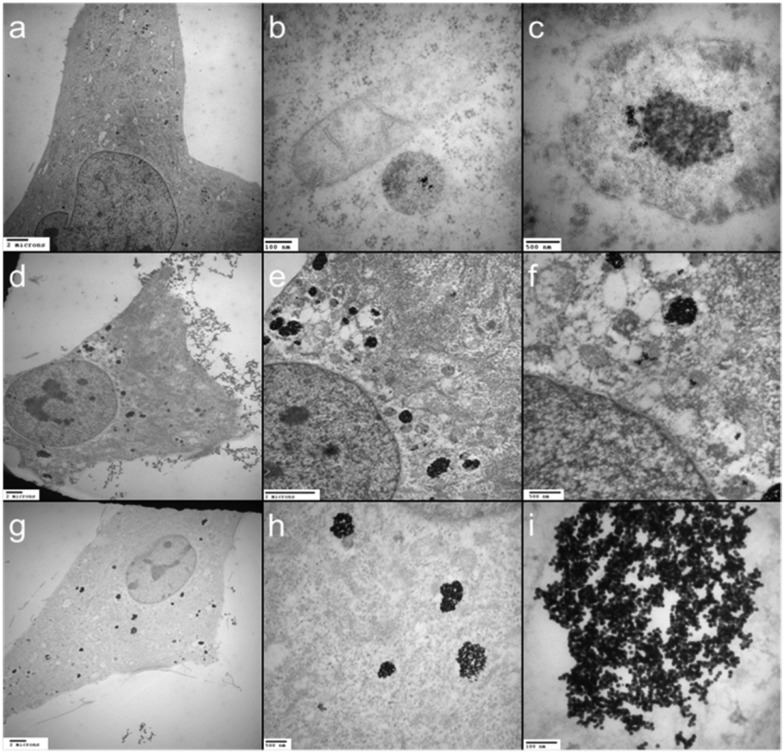
TEM images of cell-internalized Den–AuNPs of different core sizes: (a–c) 3 nm, (d–f) 12 nm, and (g–i) 17 nm. The scale bars are (a) 2 micron, (b) 500 nm, (c) 100 nm, (d) 2 micron, (e) 2 micron, (f) 500 nm, (g) 2 micron, (h) 500 nm and (i) 100 nm.

The cellular uptake and localization of Den–AuNPs were determined by TEM of cell sections (Fig. 9).^57^ The TEM images (Fig. 9a–c) show the internalization of 3 nm Den–AuNPs as aggregates, but with relatively low uptake. The cellular uptake of 12 and 17 nm Den–AuNPs (Fig. 9d–i) was significantly higher. All of Den–AuNPs were localized in vesicles throughout the cell and not found in other subcellular organelles such as the nucleus, mitochondria and golgi complex. These vesicles are seen clearly in Fig. 9, and are thought to be endosomes. The similar nanoparticle cell studies have shown that the particle clusters are uptaken by the cells *via* endocytosis.^58^ The gold concentrations added to the cells were determined by ICP-MS and the results were summarized in Table S2 (ESI†). The internalization of the 3 nm Den–AuNPs was the lowest, despite having the highest concentration in the original nanoparticle solution. The concentration of the 12 nm Den–AuNPs in the original solution was more than double compared to that of the 17 nm Den–AuNPs. Taking the concentrations into consideration along with TEM results, the cellular uptake of Den–AuNPs was largely dependent upon the size of nanoparticles, but not the concentration of gold nanoparticles with different sizes. The formation of large aggregates of the 12 and 17 nm Den–AuNPs inside the cell vesicles indicated the possible dissociation of dendrons and/or glutathione ligands from nanoparticles. The presence of aggregated gold nanoparticles inside the cells would mean potential of Den–AuNPs for therapeutic applications. The strong SP bands of aggregated citrate-capped gold nanoparticles in the NIR region after cellular uptake and their use in hyperthemia treatment for cancer treatment have recently been reported by Hainfeld, *et al.* ^59^

## 4. Conclusions

In this work, glutathione-capped gold nanoparticles of 3, 12, and 17 nm were synthesized and functionalized with dendrons resulting in dendron–conjugated gold nanoparticles. The characterization of dendron–conjugated gold nanoparticles showed no significant changes in the core size and plamon bands after dendron conjugation. Dendron-conjugated gold nanoparticles were found to be stable at pH greater than 4 and with the salt concentration of up to 100 mM NaCl. The fluorescence data showed that AMCA-labeled particles were quenched, but not DyLight 755-labeled particles. The MTT cell viability assay found that the larger particles decreased cell viability the more, and that dendron–conjugated gold nanoparticles were generally less toxic than glutathione-capped gold nanoparticles. This demonstrated the increased biocompatibility of gold nanoparticles after the conjugation of a few bis-MPA dendrons. The cellular uptake and localization of dendron–conjugated gold nanoparticles determined by TEM of cell sections showed that the cellular uptake of Den–AuNPs was largely dependent upon the size of nanoparticles.
