# Supplementary material for: Stability, cytotoxicity and cell uptake of water-soluble dendron–conjugated gold nanoparticles with 3, 12 and 17 nm cores
Source: J Mater Chem B. 2015 Jul 1;3(29):6071–80. doi: 10.1039/c5tb00608b (PMC4540059; doi:10.1039/c5tb00608b)
Supplement: Supplementary file 1 [file TB-003-C5TB00608B-s001.pdf]

Electronic Supplementary Material (ESI) for Journal of Materials Chemistry B.
This journal is © The Royal Society of Chemistry 2015

Stability, Cytotoxicity and Cell Uptake of Water-Soluble Dendron-Conjugated Gold Nanoparticles with 3, 12 and 17 nm Core

*Suprit Deol, Nisala Weerasuriya and Young-Seok Shon**

Department of Chemistry and Biochemistry, California State University, Long Beach, 1250 Bellflower Blvd., Long Beach, California, 90840, United States

ICP-MS Spectrometry. The gold concentration was quantified by ICP-MS spectrometry (Perkin Elmer 6100 Dynamic Reaction Cell (DRC) ICP-MS). A 100 µL of each sample was digested in 50 mL centrifuge tubes with 4 mL of aqua regia for 1 hour at 45 °C with the cap on. The samples were diluted with 20 mL of water and were sent for ICP-MS analysis at IIRMES in CSULB.

MALDI-TOF TOF MS Spectrometry. Den-AuNPs were decomposed with sodium cyanide prior to MALDI-TOF MS analysis (ABI 4800 Double-Focusing MALDI-TOF/TOF-MS/MS). The digestion of Den-AuNPs was done by titrating with sodium cyanide solution at 70 °C until there was no presence plasmon peak (solution was colorless). The samples were spotted on the MALDI sample plate and allowed to evaporate at room temperature, and then α-cyano-4-hydroxycinnamic acid (CHCA) matrix was applied on top of the dried samples and allowed to evaporate at room temperature.

Other Characterizations of Gold Nanoparticles. Gold nanoparticles were also analyzed by ^{1}H NMR spectroscopy, UV-VIS spectrophotometer, attenuated total reflectance-FITR (ATR-FITR) spectroscopy and transmission electron microscopy (TEM). ^{1}H NMR spectra were recorded using Bruker AC400 FT-NMR operating at 400 MHz. UV-Vis spectroscopy were obtained by using Shimadzu UV-2450 UV-spectrometer and measured from 200 to 800 nm. Infrared spectra were acquired by attenuated total reflectance (ATR) using Perkin Elmer Spectrum 100 FT-IR spectrometer. Samples for ATR-FITR were prepared on glass slides as a film by evaporating solvent under vacuum (desiccator). This process was repeated several times until the signal strength was at a detectible level. The spectra were recorded from 4500 to 450 cm^{-1}. Transmission electron microscope (TEM) images were obtained with a JEOL 1200 EX II electron microscope operating at 90 keV. Samples were prepared by placing ~25 µL of an gold

nanoparticle solution (~1 mg/mL) on a 200 mesh copper grid with formvar film. Size distribution analysis of Au nanoparticle core microscope images was executed with Scion Image Beta Release 2TM. Background subtraction was done by Rolling Ball at a set radius of 25. Measurement options were done by Ellipse Major Axis.

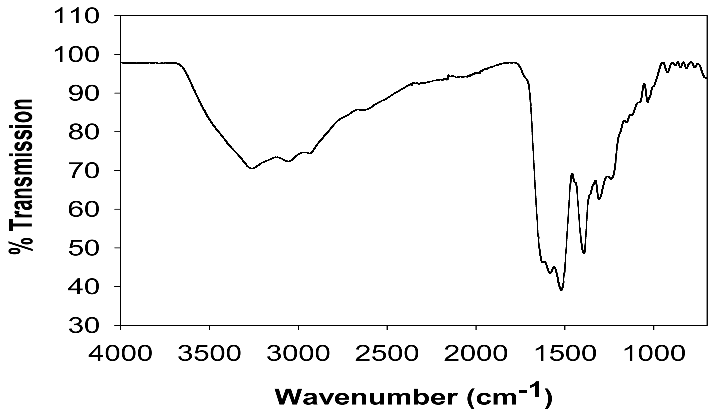

Fig. S1 FT-IR spectra of 10 nm AuNPs after the ligand exchange of citrate with reduced glutathione.

FT-IR spectra of the 10 nm AuNPs showed several new sharp absorptions at ~3000-3300 cm^{-1} after the ligand exchange of citrate with reduced glutathione (GSH). The presence of amide bonds in glutathione is also indicated by the presence of a strong absorption at ~1670 cm^{-1} in addition to carbonyl bands from either COOH (1710 cm^{-1}) or COO^- (1550 cm^{-1}) groups of glutathione.

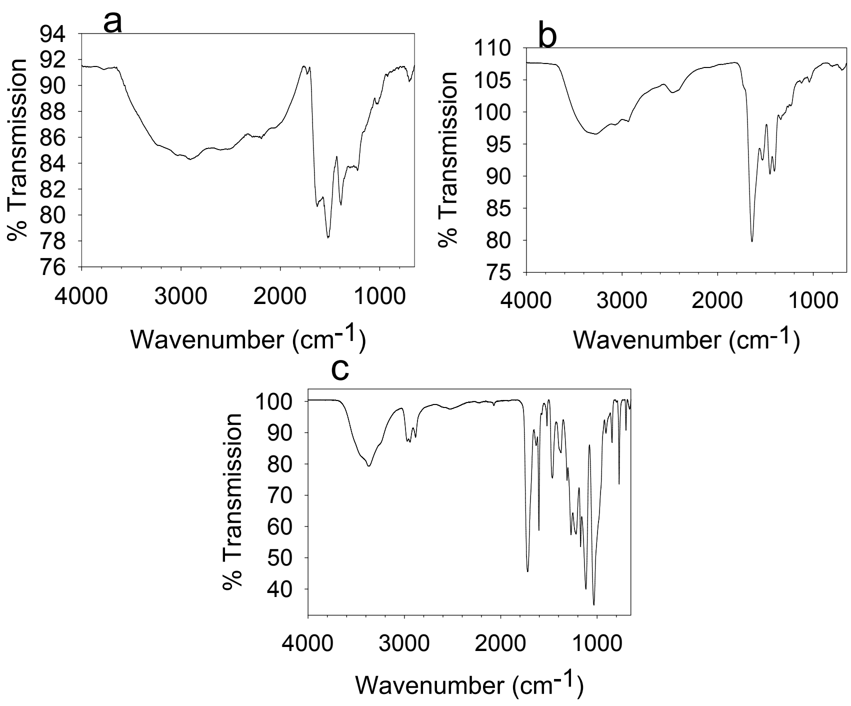

Fig. S2 FT-IR spectra of (a) 3 nm AuNPs, (b) 3 nm Den-AuNPs and (c) dendron.

FT-IR spectra of the 3 nm Den-AuNPs showed shifts in carbonyl bands at ~1600-1750 cm^{-1} after the conjugation of dendrons (Fig. S2). The formation of amide bonds after a successful coupling reaction between glutathione and dendron is indicated by the presence of a strong absorption at ~1650 cm^{-1} in place of carbonyl bands from either COOH (1710 cm^{-1}) or COO^- (1580 cm^{-1}) groups of glutathione. IR spectra of Den-AuNPs also showed a decrease in the intensity of O-H absorption at 2500-3500 cm^{-1} indicating the conversion of COOH into amide groups. This result is another supporting evidence for the conjugation of dendrons to AuNPs by the coupling reaction.

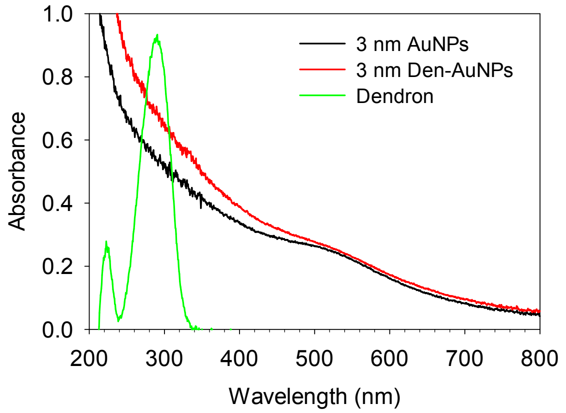

Fig. S3 UV-Vis spectra of 3 nm AuNPs, 3 nm Den-AuNPs and dendron in water.

The absorbance at 290 nm from the dendrons was too small to be clearly seen in the UV-vis spectra of the 3 nm Den-AuNPs. The UV-vis spectra of 3 nm Den-AuNPs showed a slightly broadened SP band after the conjugation of dendrons (Fig. S3). The intensity of SP band typically reflects relative average core sizes of nanoparticles and/or interactions between surrounding ligands and the surface. Therefore, the slight changes in the features and intensities of SP bands of Den-AuNPs support the dendron attachments, considering their comparable average core sizes after the coupling reaction as shown in TEM studies (Fig. S4).

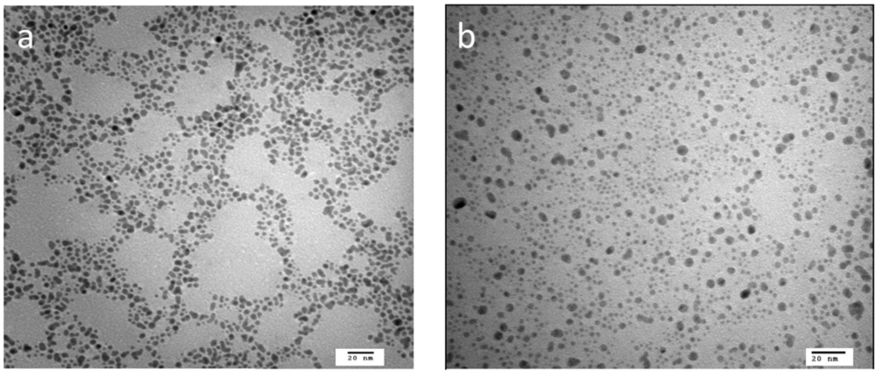

Fig. S4 TEM images of (a) 3 nm AuNPs and (b) 3 nm Den-AuNPs. The scale bars are 20 nm.

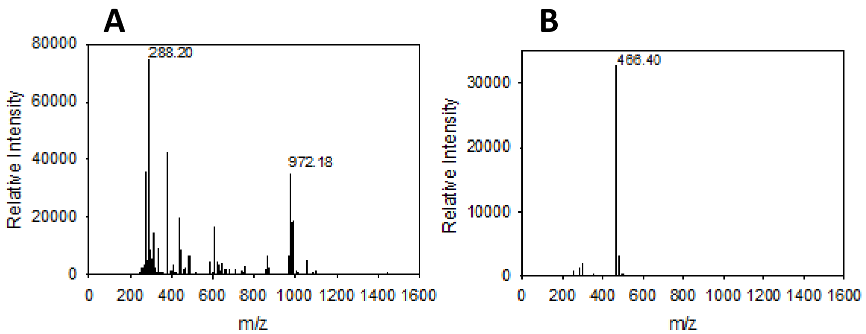

Fig. S5 MALDI-TOF/TOF mass spectrometry spectra of (a) dendron ([m+Na$^+$]=972.18) and (b) 3 nm Den-AuNPs treated with sodium cyanide.

The presence of attached dendrons was further confirmed by the MALDI-TOF/TOF mass spectra of dendron and 3 nm Den-AuNPs that are plotted in Fig. S5. The 3 nm Den-AuNPs were treated with sodium cyanide to form an Au-CN complex, liberating surface ligands. The mass spectra of 3 nm Den-AuNPs have a major m/z peak at 466.40 (Fig. S5b). This peak was assigned as a fragment of dendron formed during the sodium cyanide treatment and confirmed the presence of dendron on the surface of AuNPs. The mass spectrum of dendron itself is shown as a comparison in Fig. S5a, in which the m/z of 972.18 corresponds to the mass of dendron plus sodium ion.

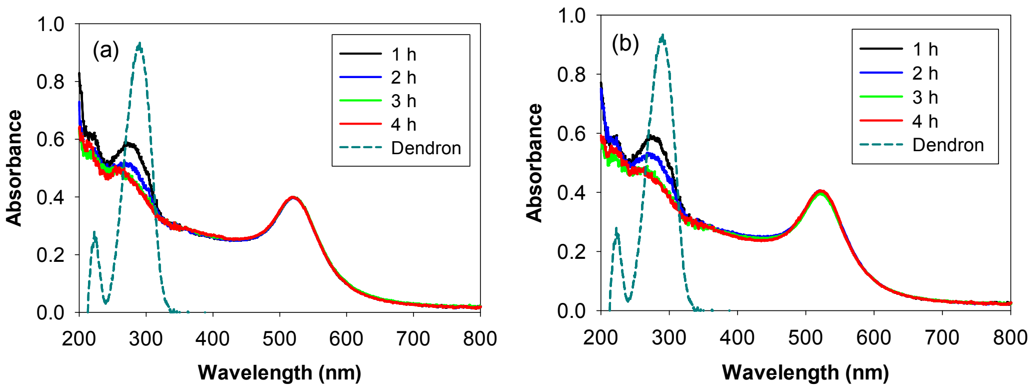

Fig. S6 UV-Vis spectra of (a) 12 nm Den-AuNPs and (b) 17 nm Den-AuNPs dialysis. Spectra were recorded after buffer changes.

The UV-vis spectra of 12 and 17 nm Den-AuNPs during dialysis are shown in Fig. S6. The spectra show that the two absorbance peaks corresponding to dendron (233 and 290 nm) decrease with subsequent buffer changes, and are still visible at the end of dialysis without any further change. The SP bands remained at around 520 nm for both Den-AuNPs without any noticeable shift, which indicates the absence of evident aggregation.

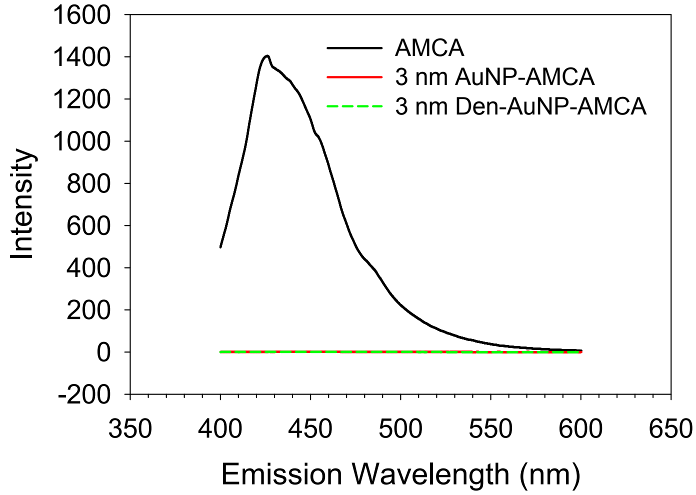

Fig. S7 Fluorescence spectra of AMCA, AMCA-functionalized gold nanoparticles (AuNP-AMCA) and AMCA-functionalized Den-AuNPs. Samples were excited at 350 nm (verify actual wavelength).

The AMCA attached to AuNPs with different core sizes (3, 12 and 17 nm) suffered from a complete quenching of fluorescence by gold core (Fig. S7). This is because the emission wavelength of AMCA at ~450 nm is close to the SP band of gold at ~520 nm.

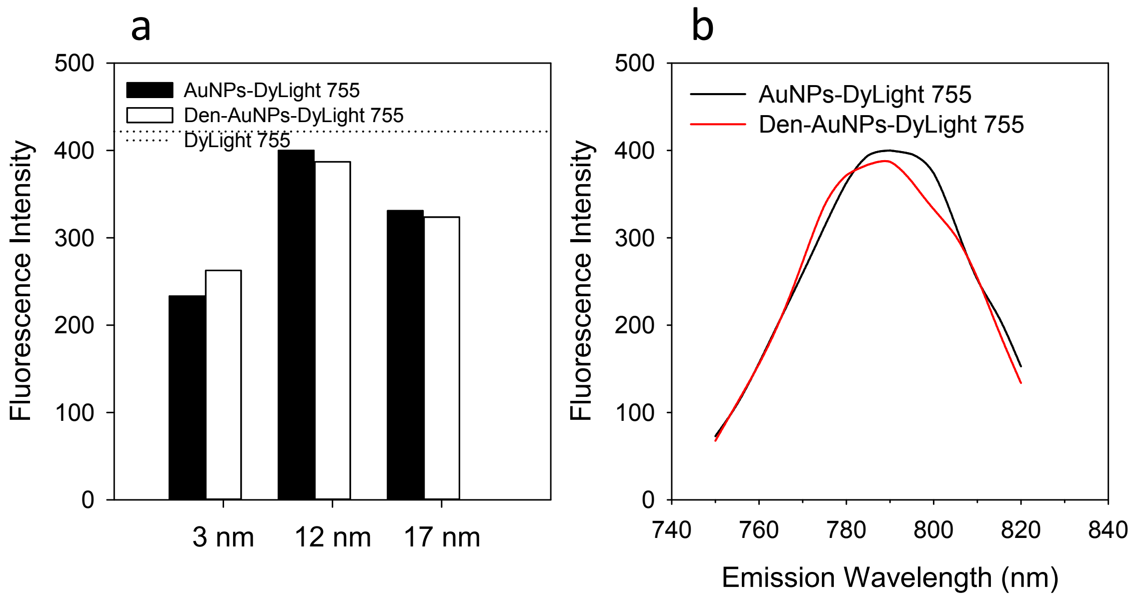

Fig. S8 (a) Fluorescence results of the DyLight 755 functionalized AuNPs and Den-AuNPs with different core sizes. (b) fluorescence spectrum of 12 nm DyLight 755 functionalized AuNPs and Den-AuNPs.

The fluorescence spectra for AuNPs-DyLight 755 and Den-AuNPs-DyLight 755 are plotted in Fig. S8. DyLight 755 NIR fluorophore exhibited strong emission properties even after attachment to gold nanoparticles with larger core sizes and was free of quenching problems previously encountered for AMCA (Fig. S8a). There is a small difference in peak emission wavelengths, which is thought to be the result of a different microenvironment created by the dendrons (Fig. S8b).

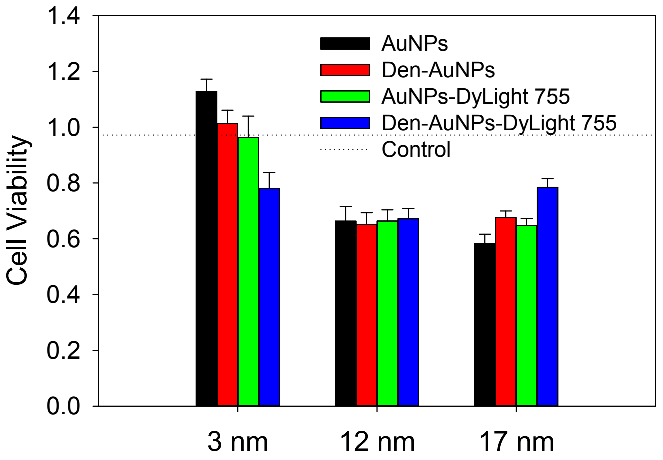

Fig. S9 *In vitro* cytotoxic assay of DyLight 755 functionalized and non-functionalized AuNPs and Den-AuNPs with different core sizes. Control (solvent) was treated with 10 mM sodium phosphate buffer, pH 7.2.

When the NIR fluorophore, Dylight-755, was used instead of AMCA, it was found that Dylight-755 did not have any significant influence over the cell viability whether they are attached to AuNPs and Den-AuNPs or they are free of any support (Fig. S9).

TABLE 1. Gold Concentration of Samples Used in Cell Toxicity Studies.

Sample	Final Concentration (ppm)
3 nm AuNPs	316.9 ± 169.2
3 nm Den-AuNPs	227.1 ± 6.0
12 nm AuNPs	9.2 ± 0.1
12 nm Den-AuNPs	6.7 ± 0.6
17 nm AuNPs	8.9 ± 0.5
17 nm Den-AuNPs	7.0 ± 0.3
3 nm AuNPs-AMCA	251.5 ± 11.6
3 nm Den-AuNPs-AMCA	274.9 ± 8.9

The nanoparticle concentrations of AuNPs and Den-AuNPs with different core sizes were different. The concentration of gold was quantified by ICP-MS (Table S1), which showed higher concentrations for 3 nm AuNPs and Den-AuNPs compared to other particles with larger core sizes.

TABLE 2. Gold Concentrations Used in Cell Uptake Studies.

Sample	Final Concentration (ppm)
3 nm Den-AuNPs	133.4 ± 5.4
12 nm Den-AuNPs	37.1 ± 1.6
17 nm Den-AuNPs	15.5 ± 0.4

The gold concentrations added to the cells were determined by ICP-MS and the results were summarized in Table S2. The internalization of the 3 nm Den-AuNPs was the lowest, despite having the highest concentration in the original nanoparticle solution. The concentration of the 12 nm Den-AuNPs in the original solution was more than double compared to that of the 17 nm Den-AuNPs. However, both Den-AuNPs resulted in similar degree of internalizations as suggested by TEM results.
